# Supplementary material for: University Students’ Mental Health and Well-Being during the COVID-19 Pandemic: Findings from the UniCoVac Qualitative Study
Source: Int J Environ Res Public Health. 2022 Jul 30;19(15):9322. doi: 10.3390/ijerph19159322 (PMC9367732; doi:10.3390/ijerph19159322)
Supplement: Supplementary file 1 [file ijerph-19-09322-s001.zip › ijerph-1846998-supplementary.pdf]

UNICOVAC
Interview topic guide

Introduction:

- Thank participant for taking part and inform them that you are recording the interview
- Introduce yourself, title of the project, funder:
  Economic and Social Research Council (ESRC), title: 'Assessing mechanisms for delivery of COVID-19 vaccines to university students'.
- Briefly outline aim of the interview: interview is a follow up to the questionnaire to gain a deeper understanding of students' pandemic experiences.
- Consent form: read out and ask participant to express consent verbally. Ask if they have any questions at this point.
- Explain that the interview will cover three broad topics: 1) the participant's experiences of COVID disease and lockdown measures; 2) their experiences of the pandemic in their local community; and 3) their media experiences, i.e. their use of various media platforms for information and guidance about the pandemic.
- Explain that the interview will last approximately one hour; participant will be asked if they wish to continue when the hour is up.

1) Experiences of COVID disease and lockdown measures:

Aim: *To give participants the opportunity to tell their story of living under COVID restrictions and establish whether they think that these restrictions are (or were) justified.*

- What is the name of your degree course and year of study?

- Thank you again for completing the questionnaire. Do you think that your views on vaccination have changed since you filled in the questionnaire? If so, what made you change your views?

- Have you had the vaccine already or will you have it very soon?
  - [*If applicable*] For you, what is [was] the single most important reason to get vaccinated against COVID?
    OR
  - [*If applicable*] What is the single most important reason for not getting the vaccine?
- Which vaccines did you previously have?
  - Have you been vaccinated against measles, mumps and rubella (MMR) and against meningitis?
  - How do you know that you have had those specific vaccines?
  - Did you use the NHS app to find out which vaccines you have had? Did you download the app?

- Have you or anyone close to you suffered from COVID?

- What is it like being a student during lockdown? What has been the worst part of it? Have there been any positive aspects to the pandemic?

- What do you think of the restrictions placed on everybody's freedom during lockdown?

- What has been your personal experience of social distancing? What do you think of masks?

- Do you think that COVID restrictions overall strike the right balance between people's health, on the one hand, and other interests (such as the economy, people's mental health and civil liberties), on the other hand? (Alternative way of phrasing this question: do you believe that COVID restrictions are proportionate or do you think that they are excessive?

- What do you think of the government's handling of the pandemic? What about other countries?

- How do you rate the COVID vaccination effort in the UK and a global level?

- Are you in favour of vaccine passports granting more freedom to fully vaccinated individuals?

2) <u>Interactions with the local community during COVID:</u>

Aim: *To give participants the opportunity to elaborate on their interactions with their local (physical and virtual) community during the pandemic, both positive and negative. Negative aspects will be explored through questions involving discrimination, stigma and racism.*

Please remind participant of University support services if they need further support, e.g. counselling. Also mention that they always talk to their personal tutor.

- Where have you mainly been living during the pandemic? What, if any, kind of solidarity or support have you received from your local community during the pandemic?

- Do you feel supported by the University, both as an organisation and as a (virtual) community in these times of pandemic?

- Can you describe any times you have experienced or witnessed stigma, discrimination, or racism?
  - Have any experiences like this occurred during the pandemic?
  - What factors or characteristics do you feel these experiences related to, for example, your ethnicity, culture, language, religion, gender, or country of birth?
  - Where did you have such experiences?

- Were these experiences interpersonal (between you and another individual) or did you feel they related to an organisational or infrastructural pattern or culture?
- How do you feel they impacted on you?

3) Media experiences of COVID:

Aim: *To give participants the opportunity to elaborate on the kind of the media they use to keep themselves informed about the pandemic and participate in COVID-related debates. Questions aim to gauge what kind of public sphere participants are engaging with.*

- Where do you usually get your information from? Do you mainly use social media or are you also using general news outlets? What are the main media for you?

- Has your use of the media changed during the pandemic? Are you spending more time on social media? Which media do you trust the most? Are there any you distrust?

- Do you actively participate in discussions online and/or generate content yourself in relation to COVID (e.g. social media posts, blogs, below-the-article comments on newspaper websites)? If yes, could you give a few examples of comments you have posted?

- Do you think that there is sufficient and sufficiently trustworthy information available in the media about COVID vaccines? Is there anything in particular about vaccines that is not sufficiently covered by the media?

- Have you ever come across fake news about the pandemic? How did you know it was false? Where did it come from?

- What is your opinion about anti-vaxxers and what they post online?

Do you have any other points that you wish to discuss? Do you have any questions?

Closing:
- Thank participant for their time
- Reminder of confidentiality
- Ask for potential to re-contact if there is there is a follow-up element in the research
